# Supplementary material for: Research progress and mitigation strategies for pod shattering resistance in rapeseed
Source: PeerJ. 2024 Oct 17;12:e18105. doi: 10.7717/peerj.18105 (PMC11491062; doi:10.7717/peerj.18105)
Supplement: Supplemental Information 1 — The PRISMA flow diagram for the systematic review detailing the database searches, the articles number of screened. [file peerj-12-18105-s001.docx]

| **Section and Topic** | **Item #** | **Checklist item** | **Location where item is reported** |
| --- | --- | --- | --- |
| **TITLE** | | |  |
| Title | 1 | Identify the report as a systematic review. | Yes |
| **ABSTRACT** | | |  |
| Abstract | 2 | See the PRISMA 2020 for Abstracts checklist. | Yes |
| **INTRODUCTION** | | |  |
| Rationale | 3 | Describe the rationale for the review in the context of existing knowledge. | Yes |
| Objectives | 4 | Provide an explicit statement of the objective(s) or question(s) the review addresses. | Yes  This review aims to bridge this gap by investigating the multifaceted factors that affect shatter resistance in rapeseed pods and exploring strategies to bolster this resistance. By improving the resilience of pods against shattering, we can effectively reduce harvest-time yield losses, ensuring increased rapeseed production and further advancing the level of agricultural mechanization in rapeseed farming. |
| **METHODS** | | |  |
| Eligibility criteria | 5 | Specify the inclusion and exclusion criteria for the review and how studies were grouped for the syntheses. | Yes  The scoping literature review conducted in this study adhered to the methodological framework outlined by [Munn et al. (2018).](https://dx.doi.org/10.1186/s12874-018-0611-x) |
| Information sources | 6 | Specify all databases, registers, websites, organisations, reference lists and other sources searched or consulted to identify studies. Specify the date when each source was last searched or consulted. | Yes  Google Scholar, Web of Science, and the Chinese National Knowledge Infrastructure；August 18, 2024 |
| Search strategy | 7 | Present the full search strategies for all databases, registers and websites, including any filters and limits used. | Yes  We employed various search engines, such as Google Scholar, Web of Science, and the Chinese National Knowledge Infrastructure, to identify pertinent articles, which were subsequently subjected to thorough screening and evaluation，We constructed four main query strings to identify relevant articles. These strings incorporated various spellings of "rapeseed," "pod shatter resistance," "agronomic measures," and "mechanized harvesting." We also included terms related to "pod length," "pod width," "number of seeds per pod," and "plant characteristics." To refine our search terms and ensure alignment with the research questions, we added another search string, focusing on keywords such as "density," "fertilizer," "plant hormone," and "gene editing." |
| Selection process | 8 | Specify the methods used to decide whether a study met the inclusion criteria of the review, including how many reviewers screened each record and each report retrieved, whether they worked independently, and if applicable, details of automation tools used in the process. | 6; Initiate a collective discourse initially, followed by individual text review and annotation |
| Data collection process | 9 | Specify the methods used to collect data from reports, including how many reviewers collected data from each report, whether they worked independently, any processes for obtaining or confirming data from study investigators, and if applicable, details of automation tools used in the process. | Methods that meet the inclusion criteria of the review；  The literature search was independently conducted by a team of six individuals, utilizing Endnote and Word text as their primary resources. Ultimately, the group members will collaboratively analyze, compare, and summarize the findings |
| Data items | 10a | List and define all outcomes for which data were sought. Specify whether all results that were compatible with each outcome domain in each study were sought (e.g. for all measures, time points, analyses), and if not, the methods used to decide which results to collect. | The findings of comparable literature studies were compared and analyzed.  The method for determining which results to collect:  1. Pre definition: Research content and keywords related to the crack resistance of rapeseed pods  2. Data availability: screening, comparing, analyzing, and summarizing to draw reliable research conclusions  3. Research objective: To identify evidence and conclusions based on the specific purpose of the study |
|  | 10b | List and define all other variables for which data were sought (e.g. participant and intervention characteristics, funding sources). Describe any assumptions made about any missing or unclear information. | This research was funded by Biological breeding major science and technology project of Sichuan Province (2022ZDZX0015) , Crop Breeding Research and Cultivation Project of Sichuan Province( 2021YFYZ0005) and Sichuan Rapeseed Innovation Team of National Modern Agricultural Industrial Technology System (SCCXTD2024-03). |
| Study risk of bias assessment | 11 | Specify the methods used to assess risk of bias in the included studies, including details of the tool(s) used, how many reviewers assessed each study and whether they worked independently, and if applicable, details of automation tools used in the process. | The discussion method; The division of labor and cooperation, the merger of similar types.;6.  This is done by searching the literature database, literature management endnote and Word. |
| Effect measures | 12 | Specify for each outcome the effect measure(s) (e.g. risk ratio, mean difference) used in the synthesis or presentation of results. | The risk ratio (RR) was utilized as a metric to assess the impact of the association between horn character and shatter resistance, The influence of transcription factors on hormone levels and the synergistic and antagonistic interactions among pre-evaluated hormone levels. |
| Synthesis methods | 13a | Describe the processes used to decide which studies were eligible for each synthesis (e.g. tabulating the study intervention characteristics and comparing against the planned groups for each synthesis (item #5)). | 1. Clarify the research objectives and questions  2. Literature search  3. Screening and preliminary evaluation  4. Full text reading and detailed evaluation  5. Extract data and create tables and graphs  For example:the characters of rape horn fruit were tabulated, and the concern between transcription factors and hormones was made into a logical mind map. |
|  | 13b | Describe any methods required to prepare the data for presentation or synthesis, such as handling of missing summary statistics, or data conversions. | Refrain from including irrelevant data and literature in the research, while ensuring that the latest relevant literature is cited. |
|  | 13c | Describe any methods used to tabulate or visually display results of individual studies and syntheses. | Identify the data sources for each study, including journals, conference papers, and databases, collect and organize the data, and finally make tables or graphs. |
|  | 13d | Describe any methods used to synthesize results and provide a rationale for the choice(s). If meta-analysis was performed, describe the model(s), method(s) to identify the presence and extent of statistical heterogeneity, and software package(s) used. | Analytic Hierarchy Process (AHP) and Data Envelopment Analysis (DEA). |
|  | 13e | Describe any methods used to explore possible causes of heterogeneity among study results (e.g. subgroup analysis, meta-regression). | Meta-regression analysis and sensitivity analysis. |
|  | 13f | Describe any sensitivity analyses conducted to assess robustness of the synthesized results. | One by one elimination method and classification elimination method. |
| Reporting bias assessment | 14 | Describe any methods used to assess risk of bias due to missing results in a synthesis (arising from reporting biases). | Shear compensation. |
| Certainty assessment | 15 | Describe any methods used to assess certainty (or confidence) in the body of evidence for an outcome. | Evaluation of the reliability of evidence sources, scientific evaluation of evidence collection methods, evaluation of data quality and completeness, and evaluation of logical consistency. |
| **RESULTS** | | |  |
| Study selection | 16a | Describe the results of the search and selection process, from the number of records identified in the search to the number of studies included in the review, ideally using a flow diagram. | 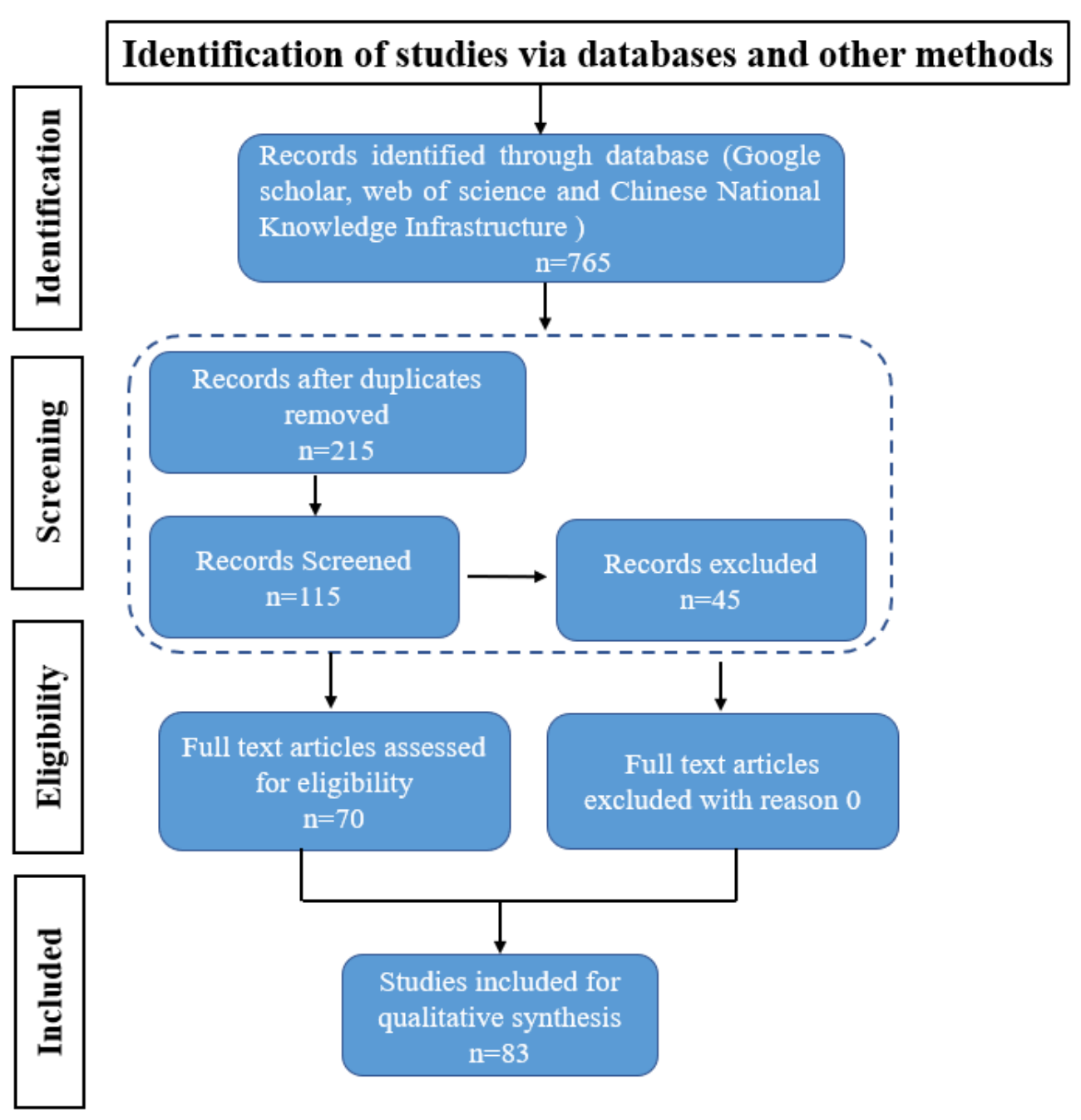 |
|  | 16b | Cite studies that might appear to meet the inclusion criteria, but which were excluded, and explain why they were excluded. | The literature was published too early, and the data were incomplete or unavailable; Other findings of serious bias were excluded. |
| Study characteristics | 17 | Cite each included study and present its characteristics. | The scoping literature review conducted in this study adhered to the methodological framework outlined by Munn et al. (2018). We employed various search engines, such as Google Scholar, Web of Science, and the Chinese National Knowledge Infrastructure, to identify pertinent articles, which were subsequently subjected to thorough screening and evaluation. Definition of research questions: Research questions guiding this systematic literature review were as follows: (1) What research approaches have been proposed to mitigate pod shatter resistance in rapeseed? (2) What are the current research achievements and future research directions in this field? |
| Risk of bias in studies | 18 | Present assessments of risk of bias for each included study. | The main contents of this review are "rapeseed," "pod shatter resistance," "agronomic measures," and "mechanized harvesting." |
| Results of individual studies | 19 | For all outcomes, present, for each study: (a) summary statistics for each group (where appropriate) and (b) an effect estimate and its precision (e.g. confidence/credible interval), ideally using structured tables or plots. | Yes  For example: Figure 2, Figure 3 and Figure 4 and Table 1 in the text. |
| Results of syntheses | 20a | For each synthesis, briefly summarise the characteristics and risk of bias among contributing studies. | Select reproducible and verifiable data and conclusions to reduce the risk of bias through comprehensive comparative analysis of the transparency of the study and strengthening data quality control. |
|  | 20b | Present results of all statistical syntheses conducted. If meta-analysis was done, present for each the summary estimate and its precision (e.g. confidence/credible interval) and measures of statistical heterogeneity. If comparing groups, describe the direction of the effect. | Mainly using systematic evaluation method. |
|  | 20c | Present results of all investigations of possible causes of heterogeneity among study results. | Methodological factors: Different studies may adopt different research designs, different varieties and different environmental conditions may lead to inconsistent research results, in addition, the selection and application of statistical analysis methods will also affect the interpretation of research results. |
|  | 20d | Present results of all sensitivity analyses conducted to assess the robustness of the synthesized results. | One by one exclusion method: After each included study is excluded one by one, the effect size is combined to observe whether the exclusion of one study has a significant impact on the overall results. |
| Reporting biases | 21 | Present assessments of risk of bias due to missing results (arising from reporting biases) for each synthesis assessed. | Qualitative and quantitative evaluations are used, and team members engage in group discussions. If the risk of report bias is considered high, caution should be exercised in interpreting the comprehensive results and pointing out possible limitations. |
| Certainty of evidence | 22 | Present assessments of certainty (or confidence) in the body of evidence for each outcome assessed. | Systematic evaluation: Determine reliable data and research conclusions based on specified evaluation criteria, systematically summarize the consistency and heterogeneity of results between different studies, comprehensively evaluate the relative importance of different influencing factors, and consider professional knowledge and practical experience in the field. |
| **DISCUSSION** | | |  |
| Discussion | 23a | Provide a general interpretation of the results in the context of other evidence. | Review existing literature related to the current evaluation results, summarize key findings from these literature, compare the current evaluation results with existing evidence, analyze the degree of consistency between them, explore possible reasons for these differences, such as research design, sample characteristics, etc., and explain the significance of the research results. |
|  | 23b | Discuss any limitations of the evidence included in the review. | 1. Limitations of research types: Different types of research have different advantages, disadvantages, and applicability, and require screening, comparison, and comprehensive analysis to draw reliable conclusions related to the topic; 2. Limitations of data collection: Some important data may not be available due to various reasons (such as privacy protection, data sharing barriers, etc.), resulting in incomplete or insufficient evidence in the review, and over time, new research continues to emerge, which can affect the timeliness of the data. |
|  | 23c | Discuss any limitations of the review processes used. | 1. Subjective limitations of review experts (standard adaptability)  2. Objective limitations of evaluation criteria (adaptability of standards)  3. External factors limitations (such as time pressure and resource constraints) |
|  | 23d | Discuss implications of the results for practice, policy, and future research. | A reduction in yield loss during mechanized harvest of rapeseed requires enhancing pod shattering resistance. The vulnerability to pod breaking is shaped by intricate internal physiological and external environmental factors. Comprehending and analyzing these complexities is vital for crafting effective mitigation strategies. This necessitates a holistic approach: genetically enhancing crack resistance, optimizing ecological conditions, meticulously managing cultivation for healthy growth, and timing harvests precisely. Further research on machine-harvest-compatible traits will bolster production efficiency, yields, and sustainable development in the rapeseed industry. |
| **OTHER INFORMATION** | | |  |
| Registration and protocol | 24a | Provide registration information for the review, including register name and registration number, or state that the review was not registered. | This review is not registered |
|  | 24b | Indicate where the review protocol can be accessed, or state that a protocol was not prepared. | The plan has not been prepared. |
|  | 24c | Describe and explain any amendments to information provided at registration or in the protocol. | No |
| Support | 25 | Describe sources of financial or non-financial support for the review, and the role of the funders or sponsors in the review. | This research was funded by Biological breeding major science and technology project of Sichuan Province (2022ZDZX0015) , Crop Breeding Research and Cultivation Project of Sichuan Province( 2021YFYZ0005) and Sichuan Rapeseed Innovation Team of National Modern Agricultural Industrial Technology System (SCCXTD2024-03);Provide page fee support |
| Competing interests | 26 | Declare any competing interests of review authors. | The authors declare no conflicts of interest. |
| Availability of data, code and other materials | 27 | Report which of the following are publicly available and where they can be found: template data collection forms; data extracted from included studies; data used for all analyses; analytic code; any other materials used in the review. | We employed various search engines, such as Google Scholar, Web of Science, and the Chinese National Knowledge Infrastructure, to identify pertinent articles, which were subsequently subjected to thorough screening and evaluation. The pertinent visual material can be located within the 83 literary sources provided in the text. |

*From:*  Page MJ, McKenzie JE, Bossuyt PM, Boutron I, Hoffmann TC, Mulrow CD, et al. The PRISMA 2020 statement: an updated guideline for reporting systematic reviews. BMJ 2021;372:n71. doi: 10.1136/bmj.n71
